# Elastic Electron Collisions with Cyanoacetylene

**DOI:** 10.1021/acsphyschemau.5c00006

**Published:** 2025-03-05

**Authors:** Victor A. S. da Mata, Giseli M. Moreira, Adevânia J. da Silva, Romarly F. da Costa, Luiz A. V. Mendes, Manoel G. P. Homem

**Affiliations:** † Departamento de Química, 67826 Universidade Federal de São Carlos, 13565-905 São Carlos, São Paulo, Brazil; ‡ Departamento de Física, Universidade Estadual do Centro-Oeste, 85040-167 Guarapuava, Paraná, Brazil; § Departamento de Física, Universidade Federal do Paraná, 81531-980 Curitiba, Paraná, Brazil; ∥ Centro de Ciências Naturais e Humanas, Universidade Federal do ABC, 09210-580 Santo André, São Paulo, Brazil; ⊥ Departamento de Ciências Exatas, Biológicas e da Terra, 28110 Universidade Federal Fluminense, 28470-000 Santo Antônio de Pádua, Rio de Janeiro, Brazil

**Keywords:** elastic electron collisions, cyanoacetylene, cross sections, shape resonances, Schwinger multichannel method, Schwinger variational method, relative flow technique

## Abstract

A comprehensive theoretical investigation involving electron collisions with cyanoacetylene is reported. Differential cross sections (DCSs), integral cross sections (ICSs), and momentum-transfer cross sections (MTCSs) for the elastic electron scattering were calculated using the Schwinger multichannel (SMC) method at the static-exchange plus polarization (SEP) level of approximation for energies ranging from 0.5 to 30 eV. The Schwinger variational method combined with Padé’s approximants (SVPA), considering static-exchange-polarization plus absorption (SEPA), was also applied for impact energies from 0.1 to 1000 eV. These results were complemented by calculations performed according to the screen-corrected independent atom model (SCIAM). Furthermore, original measurements of absolute elastic (DCSs) at 20 eV were conducted in a crossed-beam apparatus. The theoretical results display an outstanding agreement with each other and an overall agreement with the calculated cross sections available in the literature. The agreement between the calculated and measured results is quite encouraging, further validating the thoroughness of the current research.

## Graphical abstract

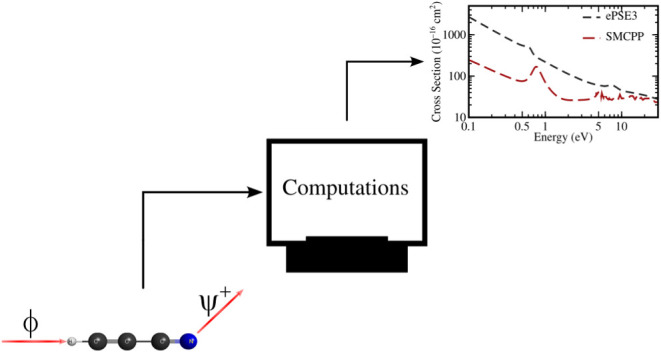

## 1 Introduction

It is consensus that the formation of matter is particularly associated with cold and dense molecular clouds in the interstellar medium (ISM) due to the medium’s interaction with ionizing fields, such as ultraviolet (UV) radiation, cosmic rays, and X-rays.^1^ The variety of molecules regarding ISM is such that up to 2021, 241 chemical species ranging from diatomic to 70-containing atoms were observed.^2^ Nowadays, the total number of detected species can be significantly larger.^3^

Also, it is well established that ionizing radiation plays a substantial role in the reaction dynamics of the ISM.^4–6^ However, electrons that arise from the primary interaction with the ionizing radiation, known as secondary electrons, can generate similar products to those observed with ionizing fields.^7^ Moreover, despite interstellar reactions being more likely to occur on the surface of the dust grains,^8^ an overall description of the molecular quantities emerges when one also accounts for the gas-phase reactivity.^9–12^ Thus, providing gas-phase data for electron collisions with molecules detected in the ISM is an issue to be clarified to deepen the understanding of electron-driven chemistry under such extreme conditions.

One crucial target within this context is cyanoacetylene (HC_3_N) [see Figure S1 of the Supporting Information (SI)], a linear molecule with a high permanent dipole moment and the smallest representative member of the cyanopolyyne series [HC_*n*_N (*n* = 3, 5, 7, ...)]. Its first detection remotes the early 1970s in the galactic radio source Sgr B2.^13,14^ Possible routes of formation of the HC_3_N molecule in the ISM medium come from ion-molecule reactions with a dissociative electron attachment (DEA) final step, as proposed by Knight *et al.* ^15^ Bates and Herbst,^16^ in turn, argued that the last step might yield HC_3_N and HNC_3_ molecules depending on their reaction branching ratio. Finally, according to Kawaguchi *et al.*,^17^ the [HC_3_N]:[HNC_3_] abundance ration in the Taurus molecular cloud (TMC-1) outlies distantly from the unit, suggesting that not only ion-molecule reaction schemes are enough to unravel the possible routes of HC_3_N formation in interstellar and circumstellar clouds, but electron collision studies as well. Studying electron-driven processes involving cyanoacetylene enhances our understanding of the ISM’s chemical complexity and reaction mechanisms.

Specifically, electron collisions with HC_3_N in the ISM can trigger several critical processes. Elastic electron scattering is important for the transport properties of the medium. Inelastic scattering can excite the rotational and vibrational states of HC_3_N, affecting its subsequent chemical reactivity. Electronic excitation may lead to radiative deexcitation of the molecule, contributing to the cloud’s emission spectrum. In the DEA process, an electron attaches to HC_3_N, leading to fragmentation and producing species such as CN^–^, HCC^–^, and CC^–^ anions. These fragments can participate in further reactions, enriching the chemical complexity of the medium. Furthermore, electron impact ionization/dissociative ionization can initiate cation-molecule reactions. The relative importance of these processes depends on the energy of the incident electrons, which in the ISM typically ranges from fractions of an eV to several eV. The cross sections required for these collision processes are crucial for modeling the electron-driven chemistry in the ISM.

The first works concerning e^–^-HC_3_N collisions are the ones of Dibeler *et al.*,^18^ Büchler and Vogt,^19^ and Harland,^20^ who performed mass spectrometric studies by measuring appearance potentials and enthalpies of formation for several ionic fragments. Posteriorly, Gilmore and Field^21^ reported experimental cross sections for the DEA of cyanoacetylene, and Ranković *et al.* ^22^ conducted electron energy-loss experiments to analyze the formation of resonant states. Theoretically, Sommerfeld and Knecht^23^ and Chourou and Orel^24^ investigated the DEA process involving this molecule with distinct approaches. Sebastianelli and Gianturco^25^ calculated elastic integral cross sections (ICSs) and probed the effect that the enlargement of the interatomic distance puts on the electron attachment. Using the *R*-matrix (RMat) method, Kaur *et al.* ^26^ computed not only elastic ICSs, but also differential (DCSs) and momentum-transfer cross sections (MTCSs). Additionally, they obtained total ionization cross sections (TICSs) operating in an amalgamation between the molecular spherical complex optical potential (MSCOP) formalism and the complex scattering potential-ionization contribution (CSP-ic). Gilmore and Field^21^ also carried out computations of TICSs. Thakar *et al.* ^27^ computed ICSs, total cross sections (TCSs), total absorption cross sections (TACSs), TICSs, and total excitation cross sections (TECSs) on electron impact with cyanoacetylene covering the 10–5000 eV energy range using the MSCOP, CSP-ic, and Binary-Encounter Bethe (BEB) methods. The most recent work of Ellis-Gibbings *et al.* ^28^ provided theoretical-experimental data involving the electron ionization of cyanoacetylene in gas phase. Their work used coincidence mass spectra recorded over the electron energy range from 50 to 200 eV to determine relative partial ionization cross sections (PICSs) and relative precursor-specific PICSs (PS-PICSs). Cyanoacetylene’s TICSs obtained using the BEB method were then used to convert their relative PICSs and PS-PICSs to absolute values.

This work reports a theoretical study of vibrationally summed elastic e^–^-HC_3_N collisions. Theoretical DCSs, ICSs, and MTCSs were obtained using the Schwinger multichannel (SMC) in the static-exchange plus polarization (SEP) approximation in the *E*_0_ = 0.5–30 eV range. Additionally, over energies ranging from 0.1 to 1000 eV, theoretical DCSs, ICSs, and MTCSs were obtained by using the Schwinger variational method combined with Padé’s approximants (SVPA). A molecular complex optical potential (MCOP) was applied to represent the e^–^-HC_3_N interaction. Further calculations above 50 eV using the independent atom model (IAM) were also performed. The atomic complex optical potential and partial-wave method were applied to obtain atomic scattering amplitudes. All the calculations were performed in a fixed-nuclei framework, *i.e.*, the degrees of freedom of nuclear motions were not considered. Therefore, the DCSs calculated using this framework are vibrationally unresolved. Despite being based on distinct physical considerations, the theoretical methods used in the present work provided results with a remarkable level of agreement, as presented in the next sections. In addition, experimental DCSs at 20 eV were also measured and reported for the first time in literature.

While previous studies have explored various aspects of electron interactions with this molecule, including ionization, DEA, and elastic scattering, none have combined experimental DCS measurements with a comprehensive theoretical treatment spanning a broad energy range (0.1 to 1000 eV). Our theoretical calculations provide a broader picture of the scattering dynamics. This combined approach, covering a wider energy range and utilizing sophisticated theoretical methods, allows for a more rigorous understanding of elastic electron scattering from cyanoacetylene.

The remainder of this paper is structured as follows: first, the experimental procedures for obtaining the elastic DCSs at 20 eV are described. Next, an outline of the theoretical methods and a summary of the computational procedures used in the calculations are provided. Finally, the results are critically analyzed and, whenever possible, compared with data available in the literature.

## 2 Experimental Procedures

Due to the commercial unavailability of cyanoacetylene, its synthesis was necessary. The synthesis of this molecule involved two steps: (1) the production of the precursor compound, propiolamide (CAS: 7341-96-0), through the reaction between ammonia and methyl propiolate (CAS: 922-67-8), and (2) the dehydration of propiolamide to obtain HC_3_N. These steps were based on the method proposed by Miller and Lemmon,^29^ with slight modifications to the procedure regarding ammonia’s preparation, which followed the approach by Homem *et al.* ^30^ The quality of the produced sample was confirmed by analysis using a commercial quadrupole attached to the high-vacuum chamber.

The experimental setup employs a crossed-beam arrangement, where the intersection of the incident electron beams and the gas defines the collision region. The setup, as well as the procedures and methodologies used, has been described in detail previously.^31^ The electron beam is generated by a commercial monochromatized electron source (Comstock EG-451), which employs a spherical sector electrostatic energy analyzer with a 36.5 mm mean radius and entrance and exit apertures of 1.0 and 0.5 mm in diameter, respectively. A two-stage electrostatic lens positioned after the exit aperture of the spherical sector allows variations in the energy and focus of the output electron beam. The scattered electrons pass through a three-element cylindrical lens, are energy analyzed by a spherical sector analyzer of the same size as the first, and are detected by a multichannel plate. The analyzer can be rotated from −10° to 110° relative to the incident beam. All surfaces directly exposed to the electron beam path are treated with colloidal graphite, and a high-permeability magnetic shield (μ-metal) covers the inner wall of the vacuum chamber, reducing the magnetic fields to less than 3 mG. The electron monochromator and analyzer systems are also enclosed within μ-metal boxes. During the measurements, the Δ*E* was kept at a value close to 180 meV (FWHM at the elastic peak). The base pressure was approximately 10^–7^ Torr, with working pressures in the range of around 1–3 × 10^–6^ Torr. The angular distributions of scattered electrons were converted to absolute DCSs using the relative flow technique (RFT),^32,33^ where the experimental elastic DCSs of N_2_ reported by Shyn and Carignan^34^ were used to normalize our data. The estimated standard deviations in the DCSs are approximately 17%.

## 3 Theory and Computational Procedures

### 3.1 Schwinger Multichannel Method

The SMC method^35,36^ and its implementations have been reviewed in the past few years^37^ and, therefore, we will only focus on describing the present calculations' important aspects. The SMC method is a variational approach to calculating the scattering amplitude which results in the following working expression

1 $$f^{\mathrm{SMC}}\left({\mathrm{k⃗}}_{f},{\mathrm{k⃗}}_{i}\right)=-\frac{1}{2\pi }\sum_{m,n}\left\langle S_{{\mathrm{k⃗}}_{f}}\right|V\left|\chi_{m}\right\rangle {\left(d^{-1}\right)}_{\mathrm{mn}}\left\langle \chi_{n}\right|V\left|S_{{\mathrm{k⃗}}_{i}}\right\rangle$$

where

2 $$d_{\mathrm{mn}}=\left\langle \chi_{m}\right|A^{\left(+\right)}\left|\chi_{n}\right\rangle$$

and

3 $$A^{\left(+\right)}=\frac{1}{2}\left(\mathrm{PV}+\mathrm{VP}\right)-VG_{P}^{\left(+\right)}V+\frac{Ĥ}{N+1}-\frac{1}{2}\left(ĤP+PĤ\right)$$

In the expressions above, {|χ_*m*_⟩} represent the (*N* + 1)­th-electron trial configuration-state functions (CSFs), which are products of target states with single-particle scattering orbitals with the proper spin-coupling. |*S*_*k⃗*_i(f)__⟩ is an eigenstate of the unperturbed Hamiltonian *H*_0_, given by the product of a target state and a plane wave with momentum *k⃗*_i(f)_; *V* is the interaction potential between the incident electron and the target; *Ĥ* ≡ *E* – *H*, where *E* is the collision energy and *H* = *H*_0_ + *V* is the scattering Hamiltonian; *P* is a projection operator onto the open-channel target space defined as

4 $$P=\sum_{l=1}^{\mathrm{open}}\left|\Phi_{l}\right\rangle \left\langle \Phi_{l}\right|$$

and *G*_*P*_^(+)^ is the free-particle Green’s function projected on the *P*-space.

The SMC calculations were performed at the SEP level of approximation. In such approximation CSFs are constructed as

5 $$\left|\chi_{\mathrm{mn}}\right\rangle =A\left|\Phi_{m}\right\rangle ⊗\left|\varphi_{n}\right\rangle$$

where |Φ_*m*_⟩ are *N*-electron Slater determinants obtained by performing single (virtual) excitations of the target, |φ_*n*_⟩ is a single-particle function and A is the antisymmetrization operator.

As mentioned above, the HC_3_N molecule has a dipole moment. In the scattering wave function expansion obtained in the SMC method, square-integrable (*L* ^2^) functions are employed, and such truncate the long-range dipole interaction. To overcome this problem, we used the Born closure procedure, which describes the interactions due to the permanent dipole potential more appropriately. This methodology has already been well discussed in several works in the literature.^37–40^ Briefly, the low partial waves in the scattering amplitude computed with the SMC method are retained up to a given $l_{\mathrm{SMC}}$ and the higher partial waves, from $l_{\mathrm{SMC}}+1$ to ∞, are included from the scattering amplitude of the dipole potential computed in the first Born approximation (FBA). The resulting expression for the scattering amplitude obtained by using the Born closure procedure is then given by

6 $$f\left({\mathrm{k⃗}}_{i},{\mathrm{k⃗}}_{f}\right)=f^{\mathrm{FBA}}\left({\mathrm{k⃗}}_{i},{\mathrm{k⃗}}_{f}\right)+\sum_{l=0}^{l_{\mathrm{SMC}}}\sum_{m=-l}^{+l}\left[f_{lm}^{\mathrm{SMC}}\left({\mathrm{k⃗}}_{i},k_{f}\right)-f_{lm}^{\mathrm{FBA}}\left({\mathrm{k⃗}}_{i},{\mathrm{k⃗}}_{f}\right)\right]Y_{lm}^{*}\left({\mathrm{k̂}}_{f}\right)$$

where $f_{lm}^{\mathrm{SMC}}$ and $f_{lm}^{\mathrm{FBA}}$ are obtained by the partial-wave expansion of the angular dependence of the outgoing wave vector *k⃗*_f_ in the SMC and the dipole FBA scattering amplitudes, respectively. The divergence of the scattering amplitude in the forward direction is avoided according to the rotational spectrum of the target molecule by making *k*_f_^2^ = *k*_i_^2^ + 2Δ*E*_rot_, where Δ*E*_rot_ is obtained as being equal to 1.88 × 10^–5^ eV in the present calculation. For HC_3_N, we use $l_{\mathrm{SMC}}=1$ from 0.15 to 0.65 eV, $l_{\mathrm{SMC}}=2$ from 0.70 to 3.50 eV, $l_{\mathrm{SMC}}=3$ from 3.60 to 3.98 eV, $l_{\mathrm{SMC}}=4$ from 4.15 to 5.60 eV, $l_{\mathrm{SMC}}=5$ from 5.80 to 7.50 eV, $l_{\mathrm{SMC}}=6$ from 8.00 to 15.00 eV, $l_{\mathrm{SMC}}=7$ from 16.00 to 22.50 eV, and $l_{\mathrm{SMC}}=8$ from 23.00 to 50.00 eV.

#### 3.1.1 SMC Computational Protocol

The molecular target ground state was described according to the Hartree-Fock (HF) approximation at the experimental equilibrium geometry.^41^ Since the SMC method deals with Abelian groups, our scattering calculations were carried out within the C_2v_ point group. In the present calculations, we employed Cartesian Gaussian (CG) basis set, generated according to a variational approximation as discussed in ref 42. To represent the carbon and nitrogen atoms, we used the 5*s*5*p*3*d* set of functions listed in Table S1 of the SI, and for the hydrogen atoms, we employed the 4*s*/3*s* basis of Dunning^43^ augmented by one *p*-type function with exponent 0.75.

To consider the polarization effects, we used the improved virtual orbitals (IVOs)^44^ to represent the particle and scattering orbitals. In the construction of the CSFs we used 9 (valence) occupied orbitals as hole orbitals, while the lowest 64 IVOs were employed as particle and scattering orbitals. Using such a choice of orbitals, the number of CSFs was 9557 for the A_1_ symmetry, 8994 for the A_2_ symmetry, and 9231 for both B_1_ and B_2_ symmetries.

### 3.2 Schwinger Variational Method Combined with Padé’s Approximants (SVPA): ePolyScat-E3 (ePSE3)

The e^–^-HC_3_N collision dynamics can be represented by a MCOP considering a static-exchange-polarization plus absorption (SEPA) approximation, *i.e.*,^45–47^

7 $$U_{\mathrm{op}}=U_{\mathrm{st}}+U_{\mathrm{ex}}+U_{\mathrm{cp}}+iU_{\mathrm{ab}}$$

where the four terms on the right-hand side are the static, exchange, correlation-polarization, and absorption interactions, respectively. This approach reduces the many-body nature of the e^–^-HC_3_N scattering to a one-particle, single-channel problem.

It is, however, difficult to solve such potential. We can avoid such complexity by using the two-potential formalism and separate *U*_op_ into a sum of two terms, such as^48^

8 $$U_{\mathrm{op}}=U_{1}+U_{2}$$

where

9 $$U_{1}=U_{\mathrm{st}}+U_{\mathrm{ex}}^{\mathrm{loc}}+U_{\mathrm{cp}}$$

and

10 $$U_{2}=U_{\mathrm{ex}}-U_{\mathrm{ex}}^{\mathrm{loc}}+iU_{\mathrm{ab}}$$

In eqs 9 and 10, *U*_ex_^loc^ represents a local model exchange potential. More details of the potentials applied in the computations are given in the next section.

The elastic DCSs are given in terms of the scattering amplitude *f*(*k⃗*_f_, *k⃗*_i_), which can in turn be expressed in terms of the *T*-matrix elements, *i.e.*,^48^

11 $$f\left({\mathrm{k⃗}}_{f},{\mathrm{k⃗}}_{i}\right)=-2\pi^{2}T_{\mathrm{fi}}=-2\pi^{2}\left\langle \phi \left({\mathrm{k⃗}}_{f}\right)\left|U_{\mathrm{op}}\right|\psi^{+}\left({\mathrm{k⃗}}_{i}\right)\right\rangle$$

where ϕ is a plane wave with wave vector *k⃗*_f_ and ψ^+^ is an outgoing spherical wave with wave vector *k⃗*_i_. We can also split *T*_fi_ as a sum of two terms, as a consequence of the two-potential formalism, to obtain

12 $$T=T_{1}+T_{2}$$

The scattering wave functions and the interaction potentials are single-center, partial-wave expanded in terms of symmetry-adapted functions for each partial wave considered (see Table 1 for further details). The elements *T*_1_ and *T*_2_ are computed, respectively, using a Runge-Kutta quadrature^49^ and Padé’s approximants.^50,51^

**Table 1:** Maximum Partial-Wave Term Used to Truncate the Scattering Equations’ Expansions

physical quantity	*l*_max_
wave functions	40
interaction potentials	80
*T*-matrix elements	20,^a^ 30^b^

a For *E*_0_ < 50 eV.

b For *E*_0_ ≥ 50 eV.

HC_3_N has an electric permanent dipole moment (μ), which generates a long-range interaction. Unfortunately, it causes the elastic DCSs to converge slowly for scattering angles close to zero. Since higher partial waves interact more effectively with the long-range dipole potential, we use a Born closure procedure to account for those higher partial waves. Briefly, the Born closure corrected scattering amplitude can be expressed as

13 $$f\left({\mathrm{k⃗}}_{f},{\mathrm{k⃗}}_{i}\right)=f^{\mathrm{FBA}}\left({\mathrm{k⃗}}_{f},{\mathrm{k⃗}}_{i}\right)+\frac{1}{k}\sum_{\mathrm{pmlh}l^{\prime }h^{\prime }}^{LL^{\prime }}i^{l-l\prime }\left(f_{\mathrm{klhl}\prime h\prime }^{\mathrm{pm}}-f_{\mathrm{klhl}\prime h\prime }^{\mathrm{FBA},\mathrm{pm}}\right)X_{\mathrm{lh}}^{\mathrm{pm}}\left({\mathrm{k̂}}_{f}\right)X_{\mathrm{lh}}^{\mathrm{pm}*}\left({\mathrm{k̂}}_{i}\right)$$

where *f* ^FBA^ and *f*_*klhl*′*h*′_^FBA, *pm*^ are the total and partial-wave expansion scattering amplitudes in the FBA, respectively, both calculated using the point-dipole potential, and *X*_*lh*_^*pm*^ symmetry-adapted functions.^52^ Such methodology is described detailedly elsewhere.^31,53^

#### 3.2.1 SVPA Computational Protocol

To represent the molecular target (HC_3_N), we performed a single-point, HF calculation with a cc-pVQZ basis set^43^ at the experimental equilibrium geometry taken from ref 41 (C_∞v_ symmetry — see Table S2 in the SI. This step was accomplished using the Gaussian 09 ^54^ software and yielded: *E*_tot_ = −168. 61513 au, μ = 4.1691 D, α_*xx*_ = 23.538 au, α_*yy*_ = 23.538 au, and α_*zz*_ = 66.808 au.

Then, the SVPA was carried out using the ePolyScat-E3 (ePSE3) suite of codes, originally developed by Lucchese *et al.* ^45,46^ Nonetheless, ePSE3 in the past was able to compute cross sections at the SEP level, with no absorption whatsoever, until we recently implemented a model absorption potential within its codes (see ref 47).

From eqs 9 and 10, *U*_st_ and *U*_ex_ were computed directly from the ground-state (*X* ^1^Σ^+^) HF/cc-pVQZ orbitals calculated herein. The model local exchange potential (*U*_ex_^loc^) was calculated from Hara’s model.^55^ We used the model correlation potential of Perdew and Zunger^56^ to calculate *U*_cp_. The α_*xx*_, α_*yy*_, and α_*zz*_ polarizabilities, determined at the HF/cc-pVQZ level of approximation, were applied in the calculation of the asymptotic part of *U*_cp_. Moreover, the model potential absorption potential of Lee *et al.* ^57^ was used to compute *U*_ab_. Cyanoacetylene’s experimental first ionization potential (11.62 eV)^58^ was applied to this model.

The expansions of the scattering equations were performed at the C_∞v_ molecular symmetry, and the cutoff parameters, given in terms of the angular momentum quantum number, are exhibited in Table 1. Convergence was achieved after up to 15 iterations.

The last step, *i.e.*, the Born closure formulas, was executed separately, as previously done by our group,^31,47,59^ since its codes are not implemented in ePSE3. The *T*-matrix elements generated from ePSE3 were applied for this purpose.

### 3.3 Independent Atom Model

Additional calculations were carried out using the screen-corrected IAM (SCIAM) proposed by Blanco and García.^60,61^ For that, an interaction potential, such as the one from eq 7, was also used at the SEPA level approximation; however, the models of Salvat *et al.*,^62^ Furness and McCarthy,^63^ Perdew and Zunger,^56^ and Staszewska *et al.* ^64^ were used to compute *U*_st_, *U*_ex_, *U*_cp_, and *U*_ab_, respectively.

## 4 Results

For clarity, the results obtained using the SVPA will be referred to simply as ePSE3 throughout the text. Additionally, for this methodology, the SEP and SEPA approximations were employed for *E*_0_ < 11.62 eV and *E*_0_ ≥ 11.62 eV, respectively. This distinction will not be explicitly stated during the discussion of the results for the reader’s convenience.

Figure 1 presents the DCSs concerning elastic e^–^-HC_3_N collisions at 2, 4, 6, and 8 eV electron energies. The SEP-SMC, SEP+Born-SMC, ePSE3, and smoothed ePSE3+Born results are displayed, along with the RMat results of Kaur *et al.* ^26^ The truncation of the partial-wave expansion in a finite number of terms leads to oscillations in the ePSE3 DCSs. The Born closure procedure should have fixed such behavior, however, perhaps due to cyanoacetylene’s too-high permanent dipole (4.1691 D), that was not possible. Therefore, we performed a visual interpolation taking the DCSs’ inflection points to eliminate those nonphysical oscillations, a methodology already accomplished elsewhere.^31,65,66^ The DCSs arising from it are called “smoothed”. For all the energies presented, the results with Born corrections largely increase toward the forward scattering, differently from those without it. In general, the DCSs tend to agree well in shape and magnitude, except for 2 eV, where the present results underestimate those of Kaur *et al.* ^26^ over almost all angles. We associate such discrepancy to differences in the energy positions of the resonant structures obtained in our calculations and those reported by the Kaur *et al*.,^26^ as discussed below.

**Figure 1:**
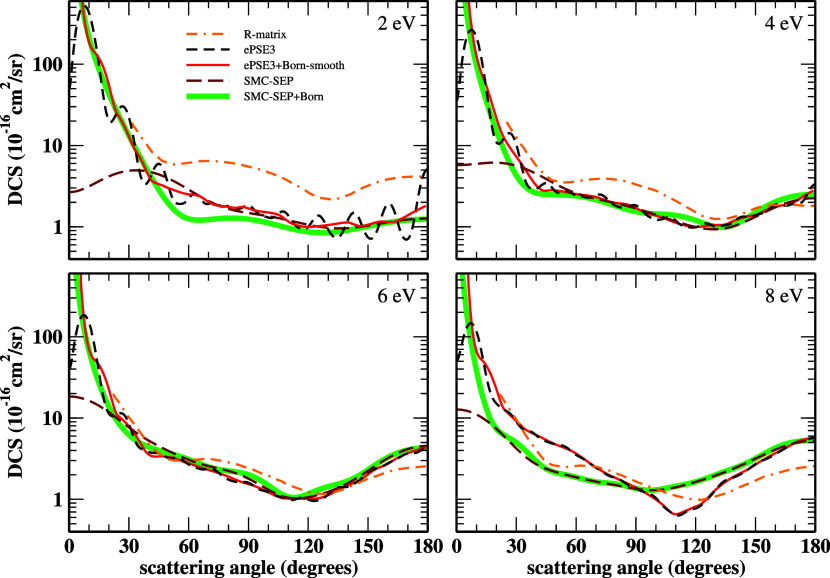
DCSs for elastic electron collisions with cyanoacetylene at 2, 4, 6, and 8 eV impact energies. See the text for further discussion.

DCSs for elastic collisions of electrons with cyanoacetylene at 10, 12, 15, and 20 eV impact energies are exhibited in Figure 2. The theoretical results from the SEP-SMC, SEP-SMC+Born, ePSE3, ePSE3+Born, alongside the RMat values of Kaur *et al.* ^26^ are also given. No interpolation of the ePSE3+Born DCSs was made for these nor higher energies since the oscillations are smoother or nonapparent. Additionally, experimental data at 20 eV are reported for the first time to the present date (see also Table S3 in the SI). Apart from the nonascending behavior over θ ≈ 0° for the DCSs without Born corrections, a good overall agreement between all the theoretical values is observed. At 20 eV, the computed DCSs agree with the experimental ones over angles smaller than 70°. More measurements, however, for other energies are yet necessary.

**Figure 2:**
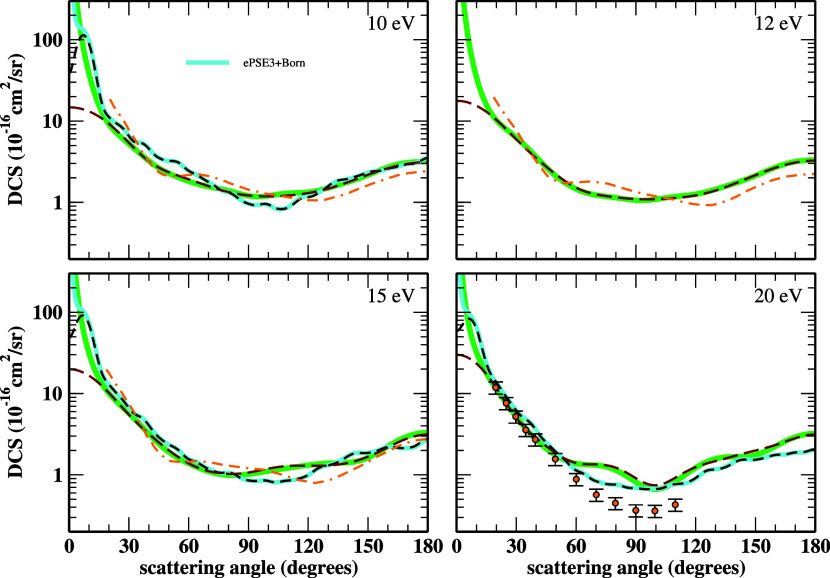
DCSs for elastic electron collisions with cyanoacetylene at 10, 12, 15, and 20 eV impact energies. The line styles are the same as in Figure 1 and the orange circles represent our experimental data. See the text for further discussion.

For energies from 30 eV and above, we present in Figures 3 and 4 the elastic DCSs of the electron scattering by HC_3_N. The SEP+Born-SMC, ePSE3, ePSE3+Born, and SCIAM results are included. We see good overall agreement in both shape and magnitude. At 30 eV, there is a good agreement between the ePSE3 and SMC DCSs. As we increase the electron energy for values above 100 eV, the electron beam interacts more efficiently with the target’s atomic cores. Thus, IAM-based methodologies describe the collision dynamics more accurately. There is a minimal discrepancy between the ePSE3 DCSs and the SCIAM DCSs over angles above 90° for energies from 150 eV and above due to ePSE3 not considering multicenter scattering as the SCIAM does, yet they present an excellent agreement.

**Figure 3:**
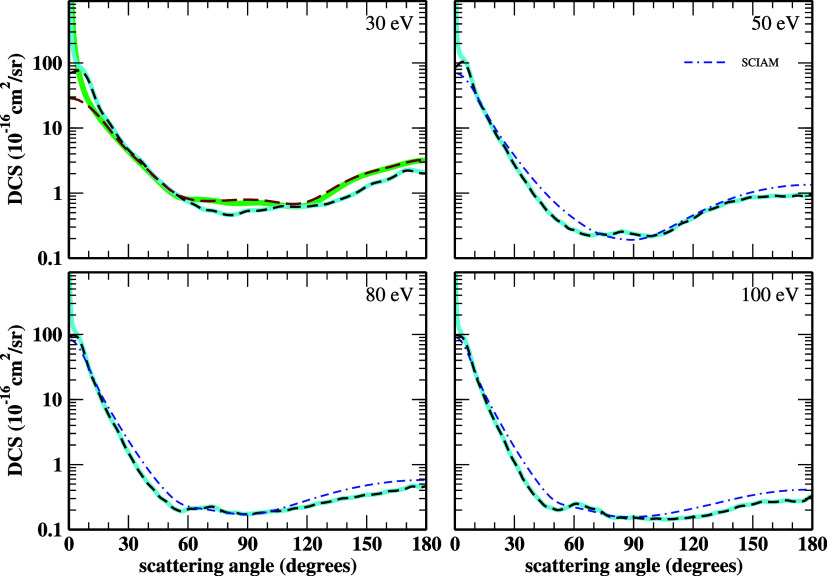
DCSs for elastic electron scattering by cyanoacetylene at 30, 50, 80, and 100 eV impact energies. The line styles are the same as in Figures 1 and 2. See the text for further discussion.

**Figure 4:**
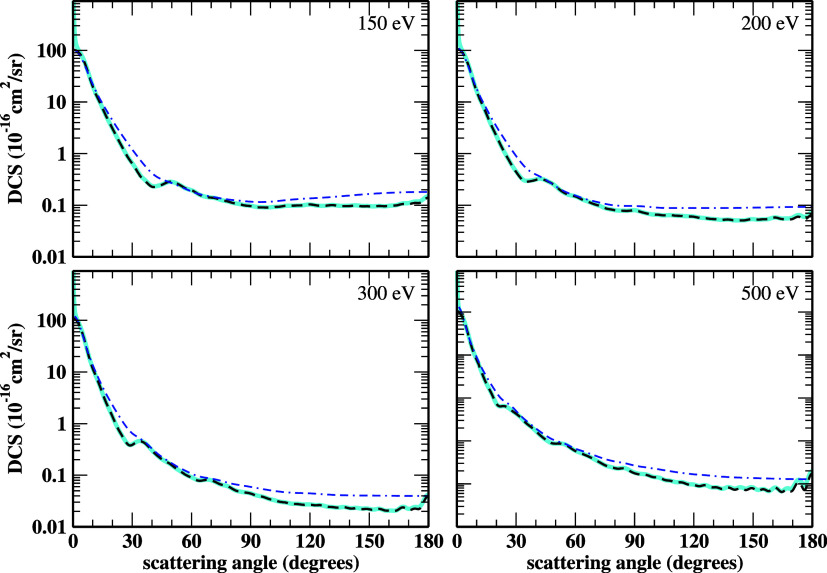
DCSs for elastic electron scattering by cyanoacetylene at 150, 200, 300, and 500 eV impact energies. The line styles are the same as in Figures 2 and 3. See the text for further discussion.

In Figure 5, the ICS (top panel) and MTCS (bottom panel) results for electron scattering by HC_3_N over the 0.1 to 30 eV energy range are presented. Regarding the ICSs, the SMC results for the SEP and SEP+Born approximations, along with the ePSE3 and ePSE3+Born ones, are included. The RMat results of Kaur *et al.* ^26^ are also included for comparison. Qualitative agreement is observed when comparing the SEP-SMC and ePSE3 ICSs. However, they deviate quantitatively, particularly for energies below 10 eV. On the other hand, a substantial improvement in the quantitative agreement is observed when the Born closure procedure is incorporated, showing that the SEP-SMC is more sensitive to this technique. The quantitative discrepancies among the ICSs computed with and without the Born correction are attributed to the ICSs being highly sensitive to the integration of the DCSs over small angles. Since the Born-corrected DCSs increase significantly over this angular region, this behavior is reflected in the ICSs, raising them to magnitudes approximately 10 times larger. It is also important to note that both the results obtained via SMC and those obtained via ePSE3 underestimate the magnitude of the results calculated through RMat.

**Figure 5:**
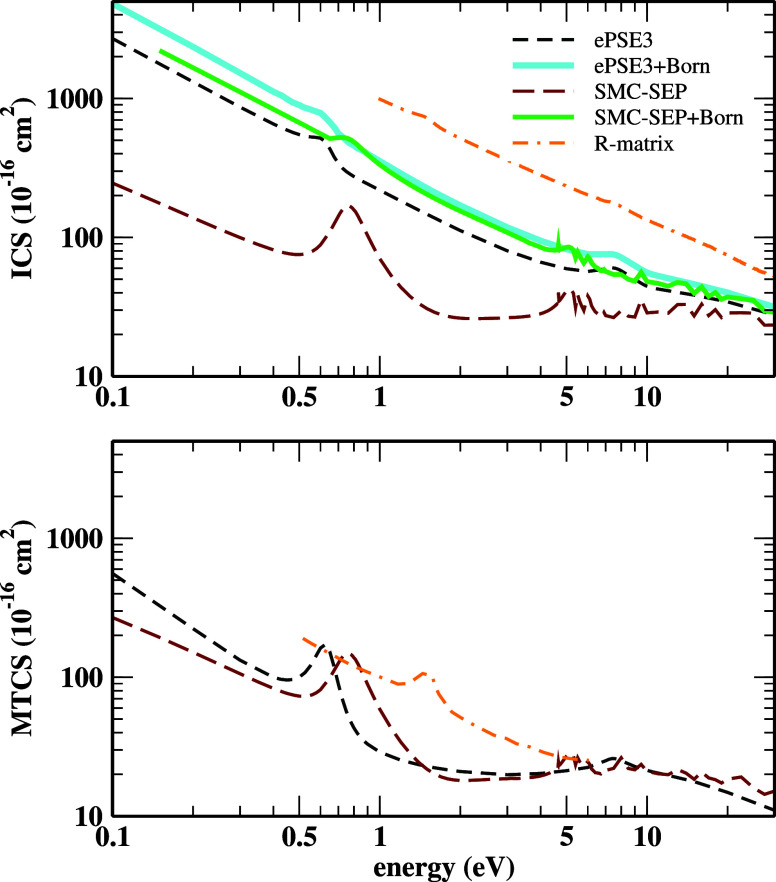
ICSs (top panel) and MTCSs (bottom panel) for elastic electron collisions with cyanoacetylene over 0.1–30 eV impact energy range. See the text for further discussion.

We can also observe the presence of shape resonances, evidenced by the ascending-descending behavior. They depict an attached electron, *i.e.*, a transient anionic state that rapidly decays into the elastic channel. Polarization effects contribute remarkably to the resonances’ positions. The positions of the center of the resonant structures obtained in the present work, jointly with others in the literature, are presented in Table 2. The first and the second resonances of the ePSE3 ICSs are shifted to lower and larger energies, respectively, compared to the SEP-SMC ICSs, due to the distinguishable strategies used to include polarization in both methodologies. Moreover, the inclusion of Born corrections masks the resonances’ intensity, especially for lower energies, as we can see from the SEP-SMC and SEP-SMC+Born ICSs, and from the ePSE3 and ePSE3+Born ICSs. Such effect happens because the dipolar background becomes very large and, as a consequence, the resonances become much smoother.^37^ However, as discussed earlier, the SEP-SMC is more sensitive than the ePSE3 to the Born closure procedure. In addition, in comparison with the results of Kaur *et al.*,^26^ similar to our findings, the authors also identified two resonant structures in the ICSs. However, the first one is much more shifted toward higher energy, which justifies the discrepancy observed in the DCS data at 2 eV, as discussed above.

**Table 2:** Position (in eV) of the Center of Resonant Structures in the Cross Sections

	π_1_^*^	π_2_^*^ + σ*
ePSE3	0.625	7.400
SEP-SMC	0.770	5.210
Sommerfeld and Knecht^23^	0.700	6.200
Sebastianelli and Gianturco^25^	1.900	8.190
RMat^26^	1.510	7.670

In Figure 5 (lower panel), our MTCSs from the SEP-SMC and ePSE3 calculations are compared with the results of Kaur *et al.* ^26^ The resonance features in the ePSE3 MTCSs are more outstanding, and their positions are better defined than in the ePSE3 ICSs. The (1 – cos θ) term, which makes the MTCSs little affected by the integration of the DCSs over angles close to zero, justifies this behavior. Hence, the magnitude of the ePSE3 and the SEP-SMC MTCSs becomes quite comparable. As the ICS results, the MTCSs of Kaur *et al.* ^26^ significantly overestimate the present results. In addition, only low-lying resonance is observed in the results of Kaur *et al.*, as the energy range of the MTCSs presented by the authors does not include the region where the second structure is located.

The ICSs can be decomposed into the ICSs comprising each symmetry that composes the target’s point group, as we can see in Figure 6, for C_∞v_’s case, calculated with ePSE3. The ePSE3 ICSs given in Figure 5, also included here, stand for the sum of all partial ICSs. It is shown in the results that the resonance located around 0.625 eV comes from the ^2^Π scattering channel while the one around 7.4 eV arises as a composition of the ^2^Σ^+^ plus the ^2^Π scattering channels. Sebastianelli and Gianturco,^25^ and Kaur *et al.* ^26^ also reached similar conclusions using distinct theoretical approaches. Other symmetries do not present any resonance feature.

**Figure 6:**
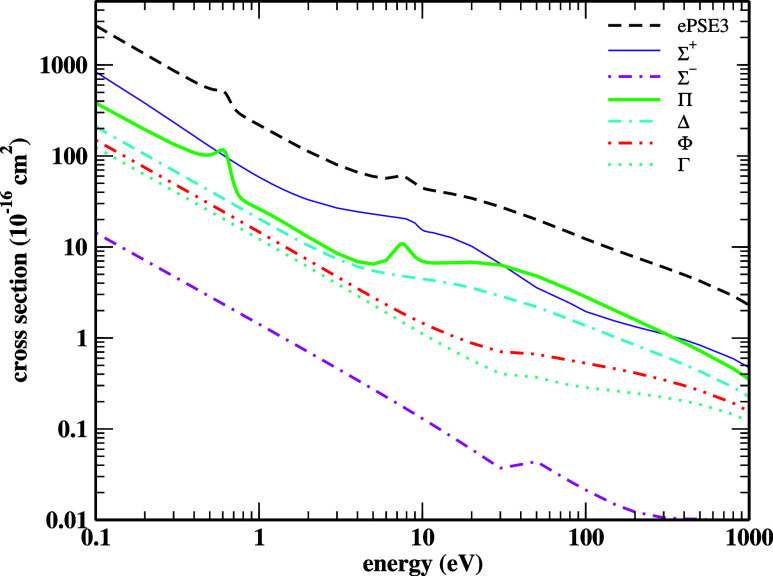
Symmetry decomposition, in the C_∞v_ point group, of the ICSs obtained with ePSE3. See the text for further discussion.

The decomposition of the ICSs in terms of the symmetries assembling the C_2v_ point group is given in Figure 7. We displayed the results from the SEP-SMC and RMat computations. The latter were reported by Kaur *et al.* ^26^ The curves reveal that the first shape resonance derives from both ^2^B_1_ and ^2^B_2_ scattering channels likewise, indicating that cyanoacetylene’s *b*_1_ and *b*_2_ orbitals are degenerate. Moreover, the second shape resonance originates from those degenerate states plus a small contribution of the ^2^A_1_ channel. The present results agree with those of Kaur *et al.* ^26^ The (B_1_, B_2_) symmetries of the C_2v_ point group correspond to the Π symmetry of the C_∞v_ point group. The same applies to the A_1_ and Σ^+^ symmetries. It shows a qualitative accordance between SMC and ePSE3 partial ICSs. The LUMO, LUMO+1, LUMO+3, LUMO+4, and LUMO+5 orbitals, which represent a good approximation of the resonant orbitals, were plotted within the C_2v_ point group and can be accessed in Figure S2 of the SI.

**Figure 7:**
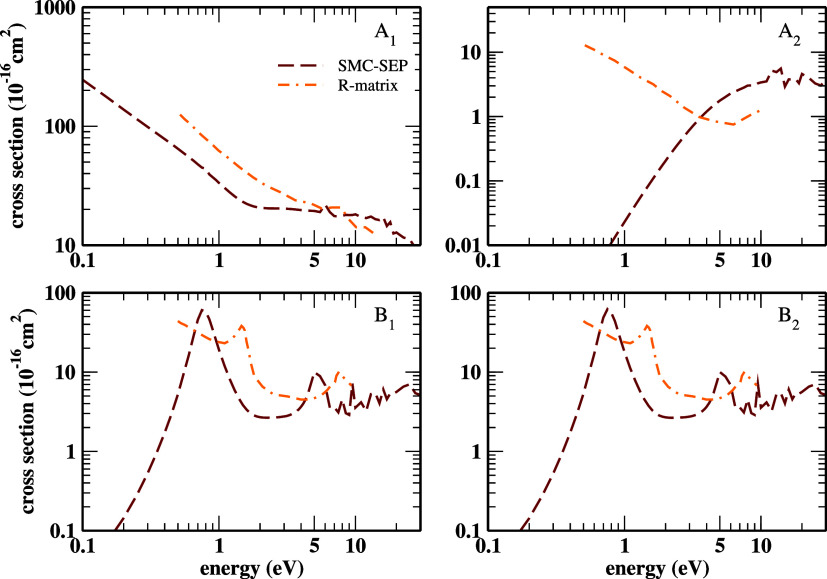
Symmetry decomposition, in the C_2v_ point group, of the ICS in the SEP approximation. We also included the RMat data reported by Kaur *et al.* ^26^ See the text for further discussion.

In summary, by integrating the use of the SMC and the SVPA methods for detailed cross section calculations, this study comprehensively analyzes elastic electron collisions with cyanoacetylene, providing results in surprisingly good agreement with each other, despite the disparate physical connotations. The critical role of polarization effects for accurate resonance prediction and of Born corrections for an adequate description of the forward-angle scattering behavior, which closely aligns with findings from previous studies, underscores the robustness of the applied methodologies. The experimental validation at 20 eV adds even more credibility to the theoretical models used to obtain the cross section results. This joint experimental and theoretical effort provides valuable insights into electron-driven chemistry in astrophysical contexts, thereby paving the way for future research that could significantly enhance our understanding of cyanoacetylene’s role in interstellar environments.

## Supplementary Material





## Notes

Details on geometry, exponents of uncontracted Cartesian Gaussian functions, molecular orbitals of HC_3_N, and raw experimental results (PDF)
DAT files with the raw theoretical results (ZIP)
