# Supplementary material for: Elastic Electron Collisions with Cyanoacetylene
Source: ACS Phys Chem Au. 2025 Mar 5;5(3):283–92. doi: 10.1021/acsphyschemau.5c00006 (PMC12123550; doi:10.1021/acsphyschemau.5c00006)
Supplement: Supplementary file 1 [file pg5c00006_si_001.pdf]

Supporting Information:

Elastic electron collision with cyanoacetylene

Victor A. S. da Mata,[*,†,⊥] Giseli M. Moreira,[‡,¶,⊥] Adevânia J. da Silva,[†]

Romarly F. da Costa,[§] Luiz A. V. Mendes,[‖] and Manoel G. P. Homem[†]

†*Departamento de Química, Universidade Federal de São Carlos, São Carlos, São Paulo, 13565-905 Brazil.*

‡*Departamento de Física, Universidade Estadual do Centro-Oeste, Guarapuava, Paraná, 85040-167 Brazil.*

¶*Departamento de Física, Universidade Federal do Paraná, Curitiba, Paraná, 81531-980 Brazil.*

§*Centro de Ciências Naturais e Humanas, Universidade Federal do ABC, Santo André, São Paulo, 09210-580 Brazil*

‖*Departamento de Ciências Exatas, Biológicas e da Terra, Universidade Federal Fluminense, Santo Antônio de Pádua, Rio de Janeiro, 28470-000 Brazil.*

⊥*Contributed equally to this work.*

E-mail: vasmata93@gmail.com

List of Figures

S1 Ball and stick model of cyanoacetylene (HC_3N) S-3

S2 Lowest unoccupied molecular orbitals (LUMOs) for HC_3N S-4

List of Tables

S1 Exponents of the uncontracted Cartesian Gaussian (CG) functions used for carbon (C) and nitrogen (N) atoms in the calculations performed with the Schwinger multichannel (SMC) method S-3

S2 Cartesian coordinates of cyanoacetylene's ground-state experimental geometry used to compute the wave function applied in ePolyScat-E3 (ePSE3) S-3

S3 Present experimental differential cross section (DCS) data (in 10^{-16} cm^2/sr) for the elastic e^--HC_3N scattering at 20 eV S-4

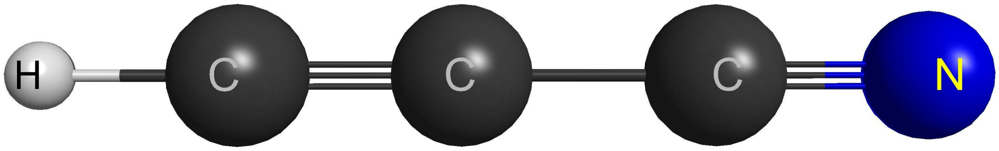

Figure S1: Ball and stick model of cyanoacetylene (HC_3N) — generated with MacMolPlt.[S1]

Table S1: Exponents of the uncontracted Cartesian Gaussian (CG) functions used for carbon (C) and nitrogen (N) atoms in the calculations performed with the Schwinger multichannel (SMC) method.

Type	C	N
s	12.496280	17.567340
s	2.470286	3.423615
s	0.614028	0.884301
s	0.184028	0.259045
s	0.039982	0.055708
p	5.228869	7.050692
p	1.592058	1.910543
p	0.568612	0.579261
p	0.210326	0.165395
p	0.072250	0.037192
d	1.794795	0.975269
d	0.420257	0.253058
d	0.101114	0.078904

Table S2: Cartesian coordinates of cyanoacetylene's ground-state experimental geometry used to compute the wave function applied in ePolyScat-E3 (ePSE3).

Atom	Coordinates (Å)[a]		
	x	*y*	*z*
N	0.0000	0.0000	1.9018
C	0.0000	0.0000	0.7413
C	0.0000	0.0000	−0.6351
C	0.0000	0.0000	−1.8409
H	0.0000	0.0000	−2.9033

[a] Taken from Ref. S2.

Table S3: Present experimental DCS data (in 10^{-16} cm^2/sr) for the elastic e^--HC_3N scattering at 20 eV. The estimated standard deviations in the DCSs are approximately 17%.

Angle (deg)	DCS
20.0	11.97
25.0	7.84
30.0	5.31
35.0	3.63
40.0	2.77
50.0	1.56
60.0	0.91
70.0	0.58
80.0	0.45
90.0	0.38
100.0	0.36
110.0	0.44

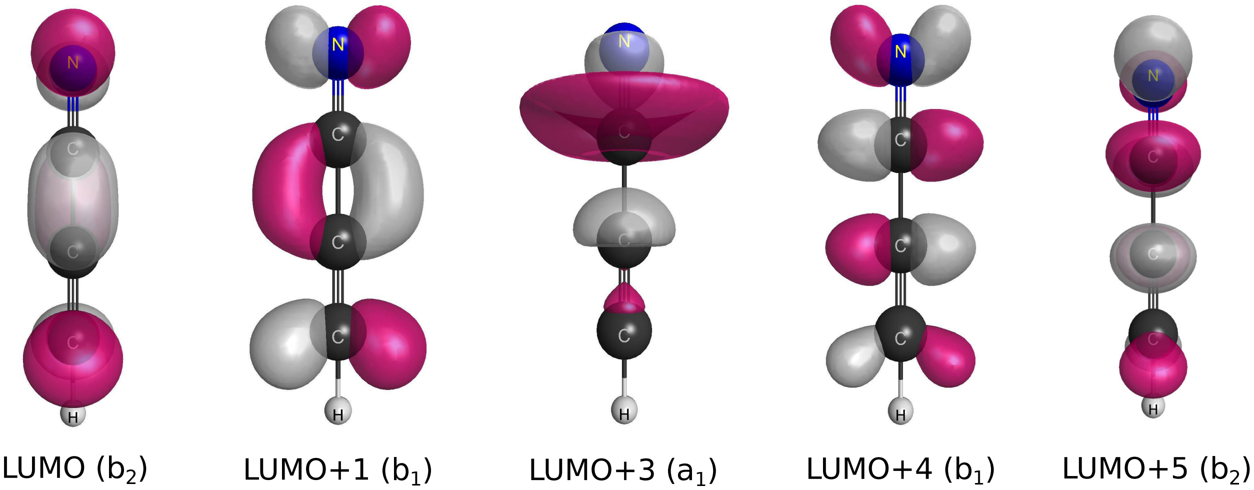

Figure S2: Lowest unoccupied molecular orbitals (LUMOs) for HC_3N (generated with MacMolPlt).[S1] The orbitals related to the B_1 and B_2 symmetries are of the π^*-type, and the orbital of the A_1 symmetry is of the σ^*-type.

References

(S1) Bode, B. M.; Gordon, M. S. Macmolplt: a graphical user interface for GAMESS. *J. Mol. Graph. Model.* **1998**, *16*, 133–138.

(S2) *Experimental data for HCCCN (Cyanoacetylene)*; Computational Chemistry Comparison and Benchmark Database Number 101, Release 19, ed. R. D. Johnson III, April, 2018.
